# Anatomic, Geographic, and Taxon-Specific Relative Risks of Carbapenem Resistance in the Health Care System of the U.S. Department of Defense

**DOI:** 10.1128/JCM.00359-16

**Published:** 2016-05-23

**Authors:** Emil Lesho, Uzo Chukwuma, Michael Sparks, Charlotte Neumann, Douglas Richesson, Robert Clifford, Sarah Gierhart, Paige Waterman, Mary Hinkle

**Affiliations:** a Antibiotic Resistance Monitoring and Research Program, Walter Reed Army Institute of Research, Silver Spring, Maryland, USA; b Antibiotic Resistance Monitoring and Research Program, EpiData Center Department Navy and Marine Corps Public Health Center, Portsmouth, Virginia, USA; c Antibiotic Resistance Monitoring and Research Program, Landstuhl Regional Medical Center, Landstuhl, Germany; d Global Emerging Infection Surveillance Section, Armed Forces Health Surveillance Branch, Silver Spring, Maryland, USA

## Abstract

Carbapenem-resistant Pseudomonas aeruginosa, Acinetobacter spp., and Enterobacteriaceae pose urgent public health threats. The differential burden, relative risks, associations with antimicrobial consumption, and temporal trends of those taxa in large, geographically diverse U.S. health systems remain under reported. Electronic records of all patients in a geographically dispersed 280-hospital managed-care system from 2005 to 2014 were reviewed. Carbapenem-resistant strains were identified based on Clinical and Laboratory Standards Institute guidelines and breakpoints. A total of 360,000 potentially carbapenem-resistant strains were identified from 14.7 million cultures (80% infecting and 20% surveillance). Isolation of bacteria overseas or isolation from the bloodstream was associated with a higher relative risks of carbapenem resistance (CR; *P* < 0.0001). Enterobacteriaceae were isolated 11 times more frequently than P. aeruginosa and Acinetobacter spp. However, compared to Enterobacteriaceae, the CR levels were 73-fold and 210-fold higher in P. aeruginosa and Acinetobacter spp., respectively. Significant differences in the relative risk of CR between taxa, anatomic, and geographic locations persisted after adjustment for other variables, the biggest differences occurring between taxa. Overall, CR rates increased for Enterobacteriaceae (*P* = 0.03) and decreased for Acinetobacter spp. and P. aeruginosa (*P* < 0.0001). These data provide a useful baseline for resistance trending and have implications for surveillance. Infections acquired overseas and bloodstream infections are particularly important areas for continued monitoring.

## INTRODUCTION

Carbapenems are one of the most important classes of antimicrobials because they remain effective against most infections increasingly caused by multidrug-resistant (MDR) and extended-spectrum β-lactamase-producing Gram-negative bacteria. Although the Centers for Disease Control and Prevention lists carbapenem-resistant Enterobacteriaceae (CRE) as an urgent public health threat (1) and recent focus has been on CRE (2, 3), Pseudomonas aeruginosa and Acinetobacter spp. are also of great concern because they frequently complicate the care of immunocompromised patients and patients injured by war or natural disasters (4–6). Furthermore, data on the burden of CR in these species, especially at the population level, remain sparse. Similarly, data are scant on whether overseas locations are associated with increased relative risk of CR, which is relevant because the number of individuals and populations (including military populations) that are mobile or displaced by conflict has increased (7, 8). Finally, selection pressure from antibiotic use is a major driver of antimicrobial resistance, with even brief exposure in the form of prophylaxis for traveler's diarrhea elevating the risk of certain types of antimicrobial resistance (9–11). However, relative risk and antimicrobial use-resistance associations at the population level or the level of an entire health system in the United States remain incompletely understood and infrequently reported.

We sought to determine here (i) the combined burden of carbapenem-resistant bacteria (CRB) (including Acinetobacter spp. and Pseudomonas aeruginosa and how that differed from resistance levels in Enterobacteriaceae) in the health care system of the U.S. Department of Defense (DOD), a large and geographically diverse managed health system; (ii) whether military treatment facilities (MTFs) located overseas or outside the contiguous U.S. (OCONUS) had an increased relative risk of isolation of a CRB compared to facilities in the contiguous United States (CONUS); (iii) whether certain anatomic sites, such as the bloodstream, had a higher risk of isolation of CRB; and (iv) whether DOD health care databases and electronic health records can be leveraged to explore antibiotic use-resistance relationships.

## MATERIALS AND METHODS

The health care system of the DOD, its beneficiaries, and detailed methods for mining electronic health care records (EHR) have been described previously (3, 12). Briefly, patients of all ages, including neonates and geriatrics, are treated in approximately 288 fixed-location facilities throughout the contiguous United States, Alaska, and Hawaii. Fixed MTFs are also located in Guam, Italy, Germany, Kuwait, Japan, South Korea, and Spain. Transient medical surgical hospitals were and/or are located in Iraq and Afghanistan. Beneficiaries include active duty, family members of active duty service members, and retirees. The average annual number of beneficiaries eligible to receive care is 9.2 million.

In terms of processes and quality, overseas facilities (except those in combat zones) are comparable to CONUS facilities in that they are held to the same requirements for Joint Commission accreditation. Similarly, clinical laboratories at fixed MTFs of the DOD are accredited by the College of American Pathologists and perform identification and susceptibility testing according to Clinical and Laboratory Standards Institute (CLSI) guidelines. Automated identification and susceptibility platform use across the DOD is fairly constant, with the Phoenix and Vitek II platforms in use by ca. 80% of hospitals and the MicroScan by ca. 20% (primarily by mobile hospitals in austere environments). This proportion of use was also stable over the entire study period. Since the health system of the DOD is geographically dispersed thought the world, it is subject to the same influences from regional and global emergences of carbapenemase-encoding genes such as bla_KPC_, *bla*_NDM_, *bla*_OXA-23_, etc., that occurred and have been documented in the literature during this study period. Soon after the start of conflicts in Iraq and Afghanistan in 2001, the DOD health system began to see significant increases in multidrug and carbapenem-resistant Gram-negative bacteria. There were no major changes to standard infection prevention and control procedures or policies during the study period.

EHR of all beneficiaries who received care at fixed MTFs were queried for all cultures that grew a target organism (Enterobacteriaceae (fermenters), Acinetobacter spp., or Pseudomonas aeruginosa (nonfermenters) from 2005 through 2014. Incidence definition was the first resistant isolate per patient per 30 day interval in a calendar year based on the CLSI guideline M39-A2 for antibiogram reporting and Hindler and Stelling (13, 14). Carbapenem resistance was defined as being resistant to ertapenem, doripenem, meropenem, or imipenem (for fermenters) or to doripenem, imipenem, or meropenem (for nonfermenters) according to prevailing CLSI and/or U.S. Food and Drug Administration susceptibility breakpoints. Not all labs can simultaneously update their breakpoints as soon as the CLSI updates them. This is an inherent and unavoidable limitation of reporting data across entire health care systems. Therefore, the specific breakpoints used were those in informational supplements M100-S16 to M100-S24 (15). To further mitigate this constraint, we also leveraged a large repository of centrally tested and characterized isolates from the DOD health system. A total of 9,000 unique (one isolate per patient per year) MDR Enterobacteriaceae, Acinetobacter spp., and P. aeruginosa underwent same-day plate testing by the referral laboratory (16, 17). The distribution of the MICs for each of the carbapenems are presented in Table 1.

**TABLE 1:** Distribution of carbapenem MICs in a representative sample of multidrug-resistant isolates from the DoD health system (one isolate per patient per year)

Organism (no. of strains)	No. of strains associated with the following carbapenem MICs									% Intermediate or resistant
	≤0.5	≤1	1	2	4	>4	8	>8	Total	
Acinetobacter spp. (2,205)										
Ertapenem	1				1	8			10	
Imipenem		646		312	62		50	1,135	2,205	56.55
Enterobacter spp. (674)										
Ertapenem	543		39	36	23	33			674	
Imipenem		470		135	52		7	10	674	30.27
Escherichia coli (4,831)										
Ertapenem	4,754		11	14	10	42			4,831	
Imipenem		4,757		27	14		11	21	4,830	1.51
Klebsiella spp. (1,222)										
Ertapenem	982		13	32	22	173			1,222	
Imipenem		1,034		21	27		27	113	1,222	15.38
Pseudomonas spp. (1,433)										
Ertapenem	1			1		7			9	
Imipenem		158		246	102		110	817	1,433	64.69

Unadjusted relative risk (RR) estimates and their 95% confidence intervals (95% CI) were calculated in Excel using formulae identical to those available elsewhere (https://www.medcalc.org/calc/relative_risk.php), which included smoothing techniques for zero counts. Adjusted RR estimates (adjusted for the categorical covariates of calendar year, specimen source, and patient location) were computed with PROC GENMOD in SAS 9.4, using Poisson regression-based methods as described previously (18). The smoothing procedure suggested by Gauvreau and Pagano (19) was utilized for calculations involving zero counts in one or more cells of the contingency table.

## RESULTS

Of 14,725,478 clinical cultures in the study time frame, 366,075 grew a target organism. We determined that 21, 8, 2, and 2% of the cultures were Acinetobacter spp., Klebsiella spp., P. aeruginosa, and E. col*i*, respectively, and the remainder were infecting cultures. Regardless of susceptibility, Enterobacteriaceae were isolated at 11 times the rate of P. aeruginosa and Acinetobacter spp. The rate of target organisms that were carbapenem resistant ranged from 1.25/1,000 organisms for E. coli to 277/1,000 organisms for Acinetobacter spp. (Table 2). The unadjusted relative risk for carbapenem resistance was 73-fold higher (95% CI = 66.6 to 80.1) in nonfermenters compared to fermenters (Table 3).

**TABLE 2:** Rates of carbapenem resistance among selected organisms

Category	No. of resistant strains (*n*) and rates/1,000 organisms															
	E. coli		K. pneumoniae		K. oxytoca		P. aeruginosa		A. baumannii		Fermenters		Nonfermenters		Total	
	*n*	Rate	*n*	Rate	*n*	Rate	*n*	Rate	*n*	Rate	*n*	Rate	*n*	Rate	*n*	Rate
Yr																
2005	19	0.92	4	1.34	1	2.61	273	93.40	203	257.29	24	1.00	476	128.23	500	17.98
2006	26	1.18	7	2.18	0	0.00	211	78.12	224	287.55	33	1.29	435	125.00	468	16.08
2007	26	1.16	8	2.51	1	2.43	235	94.61	177	306.76	35	1.35	412	134.60	447	15.41
2008	33	1.08	7	1.72	4	7.77	241	84.21	138	316.51	44	1.25	379	114.92	423	10.99
2009	48	1.47	12	2.84	2	3.75	219	79.12	98	308.18	62	1.66	317	102.72	379	9.37
2010	57	1.69	17	3.92	1	1.88	212	79.70	118	322.40	75	1.95	330	109.05	405	9.75
2011	51	1.55	25	5.52	0	0.00	225	83.49	115	363.92	76	2.01	340	112.92	416	10.18
2012	35	1.07	16	3.65	0	0.00	216	85.01	44	187.23	51	1.35	260	93.66	311	7.69
2013	35	1.28	27	7.41	2	4.37	225	101.86	23	127.78	64	2.03	248	103.81	312	9.21
2014	35	0.97	18	4.00	1	1.71	255	94.44	21	107.69	54	1.31	276	95.34	330	7.49
Specimen source																
Other	358	1.24	135	3.56	12	2.59	2,224	85.84	1,54	269.01	505	1.53	3,278	109.90	3,783	10.48
Blood	7	2.63	6	5.37	0	0.00	88	138.58	107	391.94	13	3.29	195	214.76	208	42.79
Patient location																
CONUS	324	1.25	124	3.41	11	2.44	2,154	88.01	854	261.00	459	1.53	3,008	108.41	3,467	10.59
OCONUS	41	1.27	17	6.23	1	3.41	158	76.37	307	334.06	59	1.67	465	155.62	524	13.65
Total	365	1.25	141	3.61	12	2.50	2,312	87.10	1,161	277.02	518	1.55	3,473	113.00	3,991	10.91

**TABLE 3:** Comparison of relative risks of carbapenem resistance in selected taxa

Category	Unadjusted RR	95% CI^*a*^
Organism		
E. coli	1.0	
K. pneumoniae	2.9	2.4–3.5
K. oxytoca	2.0	1.1–3.5
P. aeruginosa	69.5	62.2–77.6
A. baumannii	220.9	196.4–248.5
Fermenters vs nonfermenters		
Fermenters	1.0	
Nonfermenters	73.1	66.6–80.1

a 95% CI, 95% confidence interval.

For all taxa combined, OCONUS locations were associated with a significantly increased risk of having a resistant organism: an adjusted RR of 1.39 (95% CI = 1.26 to 1.52; P < 0.0001). Similarly, for all taxa combined, isolation from blood was associated with a significantly higher relative risk of being CR compared to all other anatomic sites: an adjusted RR of 1.94 (95% CI = 1.68 to 2.23; P < 0.0001) (Table 4).

**TABLE 4:** Adjusted and unadjusted relative risks

Category	Unadjusted				Adjusted^*a*^			
	RR	95% CI	*P*	*P* for trend	RR	95% CI	*P*	*P* for trend
Yr								
2005	1.00				1.00			
2006	0.89	0.79–1.01	0.08		0.99	0.87–1.12	0.87	
2007	0.86	0.75–0.97	0.02		1.08	0.95–1.22	0.25	
2008	0.61	0.54–0.70	<0.0001		0.92	0.81–1.05	0.21	
2009	0.52	0.46–0.60	<0.0001		0.87	0.76–0.99	0.03	
2010	0.54	0.48–0.62	<0.0001		0.93	0.82–1.06	0.29	
2011	0.57	0.50–0.64	<0.0001		0.96	0.84–1.09	0.54	
2012	0.43	0.37–0.49	<0.0001		0.77	0.67–0.89	0.0004	
2013	0.51	0.44–0.59	<0.0001		0.91	0.79–1.04	0.18	
2014	0.42	0.36–0.48	<0.0001	<0.0001	0.79	0.69–0.91	0.001	<0.0001
Specimen source								
Other	1.00				1.00			
Blood	4.08	3.55–4.69	<0.0001		1.94	1.68–2.23	<0.0001	
Patient location								
CONUS	1.00				1.00			
OCONUS	1.29	1.18–1.41	<0.0001		1.39	1.26–1.52	<0.0001	
Fermenters vs nonfermenters								
Fermenters	1.00				1.00			
Nonfermenters	73.07	66.63–80.13	<0.0001		70.71	64.44–77.59	<0.0001	
Specimen source/location								
Other/CONUS	1.00							
Blood/CONUS	1.28	1.16–1.40	<0.0001					
Other/OCONUS	3.88	3.34–4.52	<0.0001					
Blood/OCONUS	8.42	5.84–12.14	<0.0001					

a That is, adjusted for calendar year, specimen source, and patient location.

For nonfermenters alone (P. aeruginosa and Acinetobacter spp.), a higher relative risks of CR was observed for isolates recovered from the bloodstream and for isolates recovered from overseas locations. For fermenters (Enterobacteriaceae), a higher relative risk of CR was associated with blood isolation (Table 5). Even after adjusting for the other variables in the models, (adjusting for year and patient geographic location in the anatomic source model, and year and anatomic source in the geographic location model) there is an increased risk of carbapenem resistance for blood infection in both fermenters (adjusted RR = 2.21; 95% CI = 1.27 to 3.83) and nonfermenters (adjusted RR = 1.91; CI = 1.65 to 2.21). There is also an increased risk of carbapenem resistance for OCONUS locations for nonfermenters (adjusted RR = 1.43; 95% CI = 1.30 to 1.58) (Table 5).

**TABLE 5:** Characteristics associated with increased relative risk of carbapenem resistance among fermenters and nonfermenters^*a*^

Category	Fermenters						Nonfermenters					
	U-RR	95% CI	*P* for trend	A-RR	95% CI	*P* for trend	U-RR	95% CI	*P* for trend	A-RR	95% CI	*P* for trend
Yr												
2005	1.00			1.00			1.00				1.00	
2006	1.29	0.76–2.19		1.29	0.76–2.19		0.97	0.86–1.11		0.98	0.86–1.11	
2007	1.35	0.81–2.28		1.36	0.81–2.29		1.05	0.92–1.20		1.07	0.94–1.22	
2008	1.26	0.76–2.06		1.27	0.77–2.08		0.90	0.78–1.03		0.91	0.80–1.04	
2009	1.67	1.04–2.67		1.68	1.05–2.69		0.80	0.69–0.92		0.82	0.71–0.94	
2010	1.96	1.24–3.10		1.97	1.24–3.12		0.85	0.74–0.98		0.86	0.75–0.99	
2011	2.02	1.27–3.19		2.03	1.28–3.21		0.88	0.77–1.01		0.89	0.77–1.02	
2012	1.36	0.84–2.21		1.37	0.84–2.22		0.73	0.63–0.85		0.74	0.64–0.87	
2013	2.04	1.28–3.26		2.04	1.28–3.27		0.81	0.69–0.94		0.83	0.71–0.96	
2014	1.32	0.81–2.13	0.03	1.32	0.82–2.14	0.03	0.74	0.64–0.86	<0.0001	0.77	0.66–0.89	<0.0001
Specimen source												
Other	1.00			1.00			1.00			1.00		
Blood	2.16	1.24–3.74		2.21	1.27–3.83		1.95	1.69–2.26		1.91	1.65–2.21	
Patient location												
CONUS	1.00			1.00			1.00			1.00		
OCONUS	1.09	0.83–1.43		1.07	0.81–1.40		1.44	1.30–1.58		1.43	1.30–1.58	

a U-RR, unadjusted RR; A-RR, adjusted RR, meaning these values were adjusted for calendar year, specimen source, and patient location.

When species were considered individually, the relative risk of CR was higher for A. baumannii (RR = 1.27; 95% CI = 1.12 to 1.45) and K. pneumoniae (RR = 1.83; 95% CI = 1.10 to 3.03) isolated OCONUS compared to CONUS (Table S1 in the supplemental material). Upon examining anatomic sites, E. coli (RR = 2.15; 95% CI = 1.02 to 4.55), we found that A. baumannii (RR = 1.45; 95% CI = 1.19 to 1.77) and P. aeruginosa (1.61; 95% CI = 1.30 to 1.99) had a higher risk of being carbapenem resistant if they were cultured from the blood versus other body sites (unpublished data [available upon request from the corresponding author]).

## DISCUSSION

This report is notable for its size and duration, encompassing 14.7 million cultures spanning 10 years, totaling 92 million patient-years of surveillance. Significant differences in the relative risk of CR between taxa, anatomic, and geographic locations persisted after adjustment for other variables (including lactose fermentation. The most striking differences occurred between taxa. These data strongly support anecdotal observations among medical and laboratory DOD personnel, i.e., that an OCONUS location increases the relative risk of acquiring a carbapenem-resistant isolate, especially for Klebsiella and Acinetobacter spp. Also, the more serious infections (i.e., bacteremia) were more likely to be CR, particularly for E. coli, Acinetobacter spp., and P. aeruginosa. Finally, the rate of CR in this population is increasing for Enterobacteriaceae (*P* for trend = 0.03) but decreasing for Acinetobacter spp. and P. aeruginosa (*P* for trend < 0.0001).

This study has several important limitations. One limitation is that outcomes (even in the in the same study) can vary depending on what is measured for resistance and use, e.g., dichotomous, categorical, or continuous data, as well as individual drugs, drug categories, or spectrum (20). A second is that cohorts and denominators are based on relatively conservative deduplication methods, and the latest (i.e., those for 2014) lower CLSI breakpoints could not be applied across the study period. Therefore, the true burden of CR might be higher. However, one can apply the latest CLSI breakpoints to the MICs presented in Table 1 to see the effect of those breakpoints on a representative sample of 9,000 MDR isolates from the study population. The results of the population studied may not be generalizable to civilians or other health care networks, but the health care system is large and geographically diverse. It also treats patients of all ages and races and not just active-duty military. There is no guarantee that all beneficiaries who are eligible to receive care utilized fixed facilities. However, given the high costs of external health care, the majority of patients likely choose to receive care from the DOD.

Determining whether antibiotic use correlates with antimicrobial resistance is critical for designing antibiotic stewardship programs. Our ability to examine this relationship across the DOD health care system was limited. While patient-days of antibiotic use data are optimal for this analysis, it is nearly impossible to get precise patient-days or patient-years of antibiotic usage for the entire DOD health care system because even with electronic medical records, manual chart review is required to determine the exact start and stop times for each antibiotic prescription. For this reason we did not ask whether individual use correlates with patient-level resistance but instead examined how antibiotic use by an entire managed care system is related or associated with incidences of resistance.

We defined consumption by drug class as the number of antimicrobial prescriptions per antimicrobial class per patient encounter (inpatient or outpatient), meaning that, per encounter, each antimicrobial class was only counted once, irrespective of multiple prescriptions of antimicrobials within the class in that encounter. Consumption by specific drugs in a class was defined as the number of different antimicrobials per class per patient encounter. Using both Pearson product-moment and Spearman rank correlation coefficient tests, we did not detect a statistically significant positive correlation between any single drug or any combination of drugs and CR incidence for any taxa. The strongest associations (*r* > = 0.7) were for Acinetobacter spp. for all single antibiotics and combinations of drugs except carbapenems. The usage values in this study reflect population level data; therefore, the total numbers are very large. However, when *P* values are calculated based on the number of pairwise comparisons (here, 5 for each *R* value), even those with stronger *R* values (>0.70) do not reach significance. Nonetheless, the associations (or lack thereof) between antibiotic use and resistance are consistent with other studies (3, 21, 22). Furthermore, the measures used provide a baseline estimate that can be used as a crude benchmark for comparing and trending historical or future consumption in this system.

Despite these limitations, the study provides a useful baseline for future resistance trending in this population. The findings also have potential implications for surveillance, since overseas locations are important areas to continue monitoring. The findings have implications for stewardship, since fluoroquinolone and aminoglycoside use alone and in combination with carbapenems trended toward a strong association with carbapenem resistance in Acinetobacter spp. and, to a lesser extent, in E. coli (data not shown). All antibiotic classes should be used judiciously. Last, the findings have empirical treatment implications. For example, among DOD patients with bloodstream infections acquired outside the contiguous United States (especially with a preliminary microbiology report of a non-lactose-fermenting Gram-negative organism), empirical therapy should be selected with the elevated risk of CR in mind. Early consultation with an infectious diseases specialist is recommended. In conclusion, enterprise-wide surveillance for such pathogens is critical and should continue.
